# Outcome prediction of oestrogen receptor‐positive breast cancer based on a panel of oestrogen receptor‐regulated genes

**DOI:** 10.1111/his.15557

**Published:** 2025-09-12

**Authors:** Shorouk Makhlouf, Nabeelah Almalki, Amera Sheha, Nehal M Atallah, Asmaa Ibrahim, Michael Toss, Nigel P. Mongan, Emad A Rakha

**Affiliations:** ^1^ Academic Unit for Translational Medical Sciences, School of Medicine University of Nottingham Nottingham UK; ^2^ Department of Pathology, Faculty of Medicine Assiut University Assiut Egypt; ^3^ Department of Clinical Laboratory Sciences, College of Applied Medical Sciences Shaqra University Riyadh Al‐Quwayiyah Saudi Arabia; ^4^ Histopathology Department, South Egypt Cancer Institute Assiut University Assiut Egypt; ^5^ Department of Pathology, Faculty of Medicine Menoufia University Menoufia Egypt; ^6^ Histopathology Department, Faculty of Medicine Suez Canal University Ismailia Egypt; ^7^ Department of Histopathology Sheffield Teaching Hospitals, NHS Foundation Trust Sheffield UK; ^8^ Biodiscovery Institute, School of Veterinary Medicine and Sciences University of Nottingham Nottingham UK; ^9^ Department of Pharmacology Weill Cornell Medicine New York New York USA; ^10^ Department of Histopathology Nottingham University Hospitals NHS Trust Nottingham UK; ^11^ Department of Pathology Hamad Medical Corporation Doha Qatar

**Keywords:** Breast cancer, endocrine therapy, oestrogen receptor, Oestrogen receptor‐regulated genes

## Abstract

**Background:**

The response of oestrogen receptor‐positive (ER+) breast cancers (BC) to endocrine therapy (ET) is variable. ER pathway‐regulated genes have been proposed to play a role in response to ET. In this study, we investigated the prognostic and predictive impacts of the expression of key ER‐regulated genes in BC.

**Methods:**

The Cancer Genome Atlas data was used to identify differentially expressed genes (DEG) associated with ER‐positivity. Of the DEGs (1329 upregulated and 1188 downregulated genes), 21 top genes showed biological and clinical relevance to ER functions and were further investigated. Publicly available transcriptomic datasets were utilised to evaluate the clinical significance of the expression of these 21 genes. The well‐characterised Nottingham operable BC cohort was used to assess their protein expression. Genes that demonstrated prognostic significance on both levels were subsequently tested individually and in combination using multivariate Cox regression analysis.

**Results:**

Of the 21 assessed ER‐regulated genes, four genes (*PR*, *GREB1*, *AR* and *BEX1*) maintained their prognostic significance in ER+ BC at both the transcriptomic and proteomic levels. Multivariate Cox regression analyses showed that only PR and GREB1 are predictors of ET response independent of tumour grade, size or lymph node status. The combined PR‐GREB1 expression was a strong predictor of ET response.

**Conclusions:**

This study showed that when several ER‐related biomarkers were evaluated, only PR and GREB1 retained their independent prognostic significance and can be used, individually or in combination, to predict the response to ET in ER+ BC patients.

## Graphical abstract

A flow chart of oestrogen receptor‐regulated genes of predictive value in breast cancer.

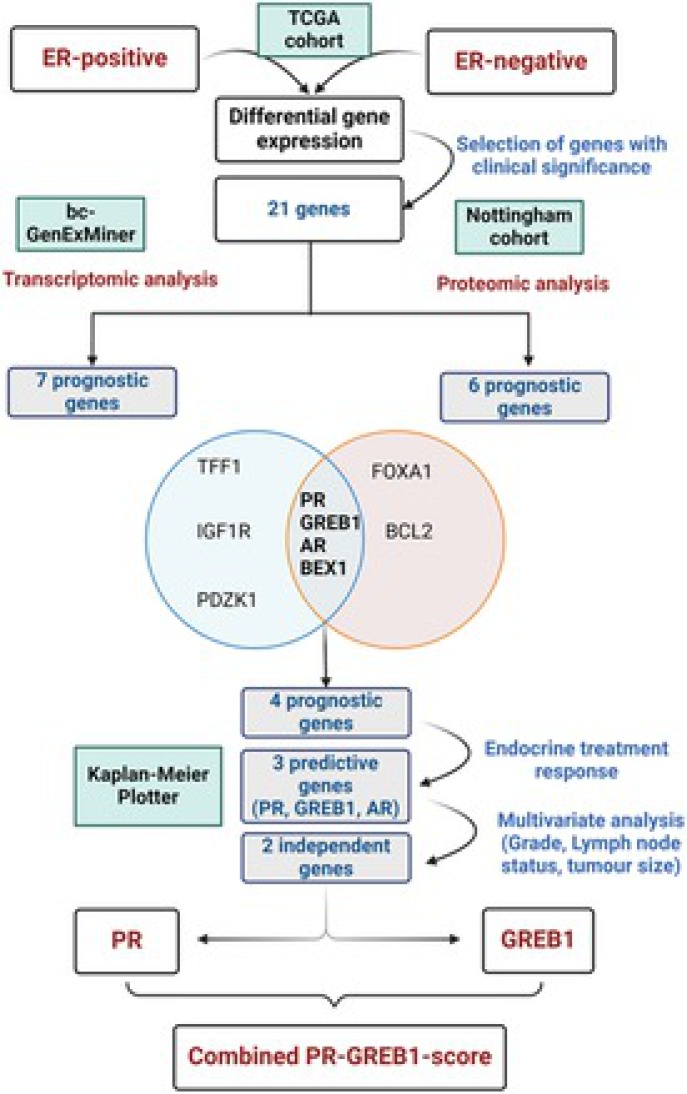

**Abbreviations**
AR: Androgen receptor
AUC‐ROC: Area under the Receiver Operating Characteristic curve
BC: Breast cancer
*BCL2*
: B‐cell lymphoma 2
BCSS: Breast cancer‐specific survival
DGE: Differential gene expression
ER: Oestrogen receptor
ERE: Oestrogen response elements
ET: Endocrine therapy
*FOXA1*
: Forkhead box protein A1
GDC: Genomic Data Commons
GREB1: Growth Regulating Oestrogen Receptor Binding 1
HER2: Human epidermal growth factor receptor 2
IHC: Immunohistochemical
METABRIC: Molecular Taxonomy of Breast Cancer International Consortium
PR: Progesterone receptor
TCGA: The Cancer Genome Atlas
*TFF1*
: Trefoil Factor 1
TMA: Tissue microarray

## Introduction

Breast cancer (BC) is the most common cancer affecting females worldwide, accounting for over 2.3 million cases every year.^1^ BC is a heterogeneous disease with variable morphology, molecular profiles, prognosis and response to therapy.^2^ Over 70% of BCs express oestrogen receptor (ER+), which harbours a favourable prognosis and is a strong predictor of response to endocrine therapy (ET).^3^ However, a proportion, up to 50% of ER+ BCs, fail to respond to ET,^4^, ^5^, ^6^, ^7^ which indicates that the ET response cannot be predicted based on ER expression alone^8^ and that other ER‐related genes that are likely to play a role in ET response can be used to refine the predictive stratification of ER+ patients to ET.

Oestrogen‐activated ERs bind to oestrogen response elements (ERE) on target genes, initiating a complex sequence of events involving epigenetic regulators that alter histone structures to enable transcription.^9^ ER‐signalling largely depends on the activity of these direct ER‐target genes and downstream secondary targets that contribute to various cellular and metabolic functions, transcription, translation and cell cycle regulation.^10^ Identifying key ER‐target genes involved in ET response could provide a better understanding of the transcriptional and biological function of ER and could refine the prediction of response to ET^11^ and improve our understanding of treatment resistance.^12^

Several genes such as forkhead box protein A1 (*FOXA1*),^13^, ^14^ trefoil Factor 1 (*TFF1*)^15^ and B‐cell lymphoma 2 (*BCL2*)^16^ have been identified as ER‐regulated. However, most of these genes have not been proven to be highly specific to ER response as the expression of these genes is also regulated by alternative pathways independent of the direct ER‐signalling pathway.^17^ Additionally, some of these genes lack sufficient validation in large cohorts. Therefore, the identification and characterisation of key gene(s) that can refine the prediction of response to ET when several genes are evaluated together remains to be defined.^17^, ^18^

In this study, we aimed to investigate the prognostic significance of the well‐known ER‐target genes and determine their predictive ability for ET responsiveness using transcriptomic and proteomic analyses.

## Materials and Methods

### Study Cohorts

#### The cancer genome atlas (TCGA) data

Gene expression data was accessed for 855 primary BC tumour specimens from the publicly available TCGA database via the Genomic Data Commons (GDC) data portal (https://portal.gdc.cancer.gov/). Deidentified clinical data were downloaded from https://www.cbioportal.org/. Differential gene expression analysis (DGE) was carried out after dividing the cases according to ER status into two groups (ER+ and ER‐). The analysis was performed using iDEP.96 (an integrated web application for differential expression and pathway analysis of RNA‐Seq data).^19^ The gene expression file was uploaded to iDEP, values were normalised and the DESeq2 method was selected. A minimum fold change = 2 and adjusted p‐value (FDR) cutoff <0.05 were set for analysis. Normalised gene counts were used for correlation analyses. Heatmaps and data visualisation were also presented.

#### Breast cancer gene‐expression miner (bc‐GenExMiner)

The bc‐GenExMiner online platform for gene prognostic and expression correlated analyses in BC^20^, ^21^ was utilised. All available RNAseq data (*n* = 4,421) were converted to a common scale (median equal to 0 and standard deviation equal to 1^b^) to create pooled cohorts and were then analysed. This cohort was used for correlation analysis and to test the prognostic significance of studied genes in ER+ BC, in terms of overall survival (OS) and disease‐free survival (DFS). However, the treatment data for this cohort was not available.

#### Kaplan–Meier plotter

RNAseq data of 1444 ER+ ET‐treated BC was explored using Kaplan–Meier Plotter, an online survival analysis tool integrating gene expression data and clinical data.^22^ A scale normalisation was performed to reduce batch effects, setting the mean expression of the overlapping probes to 1000 in each array. All patients in this dataset were ET‐treated and were utilised to investigate the predictive significance of the studied genes.

#### Nottingham cohort

A well‐characterised BC cohort of patients (*n* = 4,222)^23^ presented to Nottingham City Hospital, Nottingham, UK was investigated and used for immunohistochemical (IHC) assessment. Clinicopathological parameters, treatment regimens and follow‐up data were collected from the data registry (Table S1). BC‐specific survival (BCSS) was calculated as the time from the initial diagnosis to the time of BC‐related death. The mean patient follow‐up was 163 months.

Data on ER, progesterone receptor (PR) and human epidermal growth factor receptor 2 (HER2) were retrieved.^24^, ^25^, ^26^, ^27^, ^28^ Hormone receptor and HER2 scoring were assessed according to the UK guidelines and the American Society of Clinical Oncology and College of American Pathologists (ASCO CAP) guidelines.^29^, ^30^

The cohort included both ER+ and ER‐ BC for comparison. ER was positive in 77% of the cohort. In the entire cohort, 40% of patients were treated with ET, 16% received only chemotherapy and 10% received both. Within the cohort of ER+ patients, 49% received exclusive ET and 12% received both ET and chemotherapy. The cohort also included older cases that did not receive adjuvant therapy. The treatment protocols are based on ER status, Nottingham Prognostic Index, menopausal status and patient tolerance.^23^

This study was approved by the Yorkshire & the Humber ‐ Leeds East Research Ethics Committee (REC Reference: 19/YH/0293) under the IRAS Project ID: 266925. Data collected were fully anonymised. All procedures performed in the study were in accordance with the Declaration of Helsinki.

#### Molecular taxonomy of breast cancer international consortium (METABRIC)

Data on ER+ ET‐treated BC patients from the METABRIC cohort^31^ (n = 736) was used as an external validation cohort. The METABRIC study was carried out on fresh‐frozen primary BC samples obtained from tumour banks in the United Kingdom and Canada. RNA was isolated from these samples for transcriptional profiling. The gene expression raw data obtained was pre‐processed and normalised as previously explained.^31^

### Oestrogen Receptor‐Regulated Biomarkers Panel

Of the extensive list of DEGs, 21 genes were selected from the top genes. Those genes have been previously suggested to be ER‐regulated, exhibited a significant association with ER expression and showed prognostic/predictive value.^32^, ^33^, ^34^, ^35^, ^36^, ^37^, ^38^, ^39^, ^40^, ^41^, ^42^, ^43^, ^44^, ^45^, ^46^, ^47^ The Nottingham cohort provided data on their protein expression, including details of their staining and scoring, which were previously published^42^, ^43^, ^48^, ^49^, ^50^, ^51^, ^52^, ^53^, ^54^, ^55^ (Table S2 and Figure S1). The expression data for the GREB1 protein was unavailable. As a result, we conducted an IHC staining for GREB1, the details of which are outlined below.

### Immunohistochemistry

IHC staining was carried out on tissue microarray (TMA) sections. Four‐micrometer sections of TMA blocks were cut and a Leica XL autostainer (Leica Biosystems, Milton Keynes, UK) was used. Sections were deparaffinised and microwave antigen retrieval was performed using citrate buffer (pH 6) for 20 min. Anti‐GREB1 rabbit polyclonal (ab72999, Abcam) primary antibody incubation was applied for 1 h at room temperature, at a dilution of 1/100. Novolink polymer detection kit (Leica Biosystems, Newcastle, UK, RE7230‐K) was used for staining visualisation. Counterstaining was performed with Mayer's haematoxylin. Positive and negative tissue controls (human tonsil and kidney) were included.

Biomarker scoring followed REMARK (reporting recommendations for tumour marker prognostic studies) criteria. Stained sections were scanned using Panoramic 250 Flash III (3D Histech, Budapest, Hungary) with high‐resolution whole‐slide images. Each TMA slide was assessed blindly to the clinicopathological data. The percentage of positive cytoplasmic staining of tumour cells was assessed by one observer and validated by another observer who double‐scored 20% of the cases. An intraclass correlation coefficient (ICC) of 0.8 was obtained.

### Statistical Analysis

Statistical tests were performed using the statistical package for the social sciences (IBM SPSS v28). The Chi‐square test was used to test the correlation between categorical variables. Gene expression count correlations were tested by Pearson's rank correlation coefficient. ICC was utilised to evaluate the interobserver concordance. Following the established ER‐positive threshold, biomarker protein expression was classified based on the presence (≥1%) or absence of IHC staining. Gene counts were categorised into low and high groups based on the median expression. The accuracy of ER‐regulated biomarkers in predicting ER positivity was assessed using the area under the Receiver operating characteristic (ROC) curve (AUC‐ROC). Kaplan– Meier method and log‐rank test were applied to predict the outcome. Multivariate Cox Regression analysis with hazard ratios and confidence intervals was used to test for prognostic independence. GraphPad Prism 9.5.1 was used for graphical presentation. A *P*‐value (two‐tailed) of <0.05 was considered significant in all tests.

## Results

### Transcriptomic Analysis

#### Differentially expressed genes

Differential gene expression analysis of the TCGA cohort identified 1329 over‐expressed genes and 1188 down‐regulated genes in ER+ BCs compared to ER‐ BCs, which passed the preset significance level. Of the over‐expressed genes, a set of 21 ER‐regulated genes was selected for further investigation (Figure S2). Table S3 demonstrates the DGE analysis metrics of the genes further evaluated in this study.

#### Correlation to ESR1

**Figure 1:**
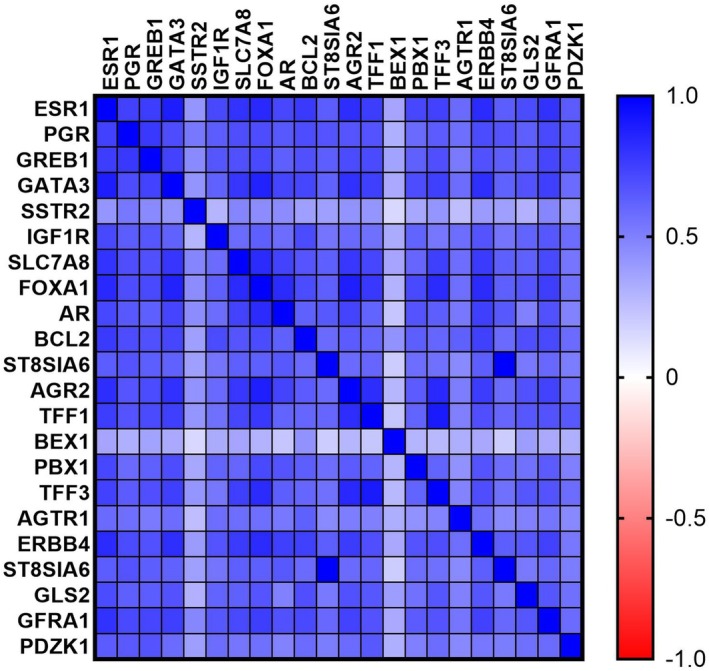
Correlation matrix of gene expression values of ESR1 and ER‐regulated genes extracted from The Cancer Genome Atlas BRCA Dataset.

A significant positive linear correlation (*p* < 0.001) was found between each of the 21 ER‐regulated genes and *ESR1* expression as continuous normalised RNA‐seq count, while variable correlations were seen between *ESR1* expression and these genes' categorical groups (Table 1). A correlation matrix for *ESR1* and ER‐regulated gene expression values is presented in Figure 1.

**Table 1:** Correlation between each oestrogen receptor (ER)‐related gene and *ESR1* as continuous expression values in RNA‐seq data extracted from bc‐GenExMiner and as stratified by median values in the cancer genome atlas (TCGA) data

RNA‐seq data (bc‐GenExMiner) (*n* = 4,421)		TCGA (n = 854)			
Gene symbol	Correlation to *ESR1* ^a^	*P*‐value	High gene/high *ESR1 N (%)*	High gene/low *ESR1 N (%)*	*P*‐value^b^
*GATA3*	0.84	<0.001	322 (75)	105 (25)	<0.001
*FOXA1*	0.75	<0.001	212 (50)	215 (50)	<0.001
*SLC39A6*	0.74	<0.001	324 (76)	103 (24)	<0.001
*ERBB4*	0.73	<0.001	305 (71)	122 (29)	<0.001
*BCL2*	0.71	<0.001	307 (72)	120 (28)	<0.001
*GFRA1*	0.70	<0.001	313 (73)	114 (27)	<0.001
*IGF1R*	0.67	<0.001	310 (73)	117 (27)	<0.001
*AGR2*	0.66	<0.001	295 (69)	132 (31)	<0.001
*SLC7A8*	0.66	<0.001	298 (70)	129 (30)	<0.001
*AR*	0.63	<0.001	279 (65)	148 (35)	<0.001
*PR*	0.62	<0.001	289 (68)	138 (32)	<0.001
*GREB1*	0.61	<0.001	288 (67)	139 (33)	<0.001
*TFF1*	0.59	<0.001	276 (65)	121 (35)	<0.001
*TFF3*	0.59	<0.001	276 (63)	160 (37)	<0.001
*GLS2*	0.55	<0.001	290 (68)	137 (32)	<0.001
*PBX1*	0.55	<0.001	299 (70)	127 (30)	<0.001
*ST8SIA6*	0.55	<0.001	278 (65)	147 (35)	<0.001
*PDZK1*	0.51	<0.001	287 (67)	140 (33)	<0.001
*AGTR1*	0.44	<0.001	258 (61)	167 (39)	<0.001
*BEX1*	0.26	<0.001	241 (53)	212 (47)	0.04
*SSTR2*	0.23	<0.001	234 (54)	198 (46)	0.01

^a^ Pearson's rank correlation coefficient. ^b^Chi‐square test.

#### Survival analysis

**Figure 2:**
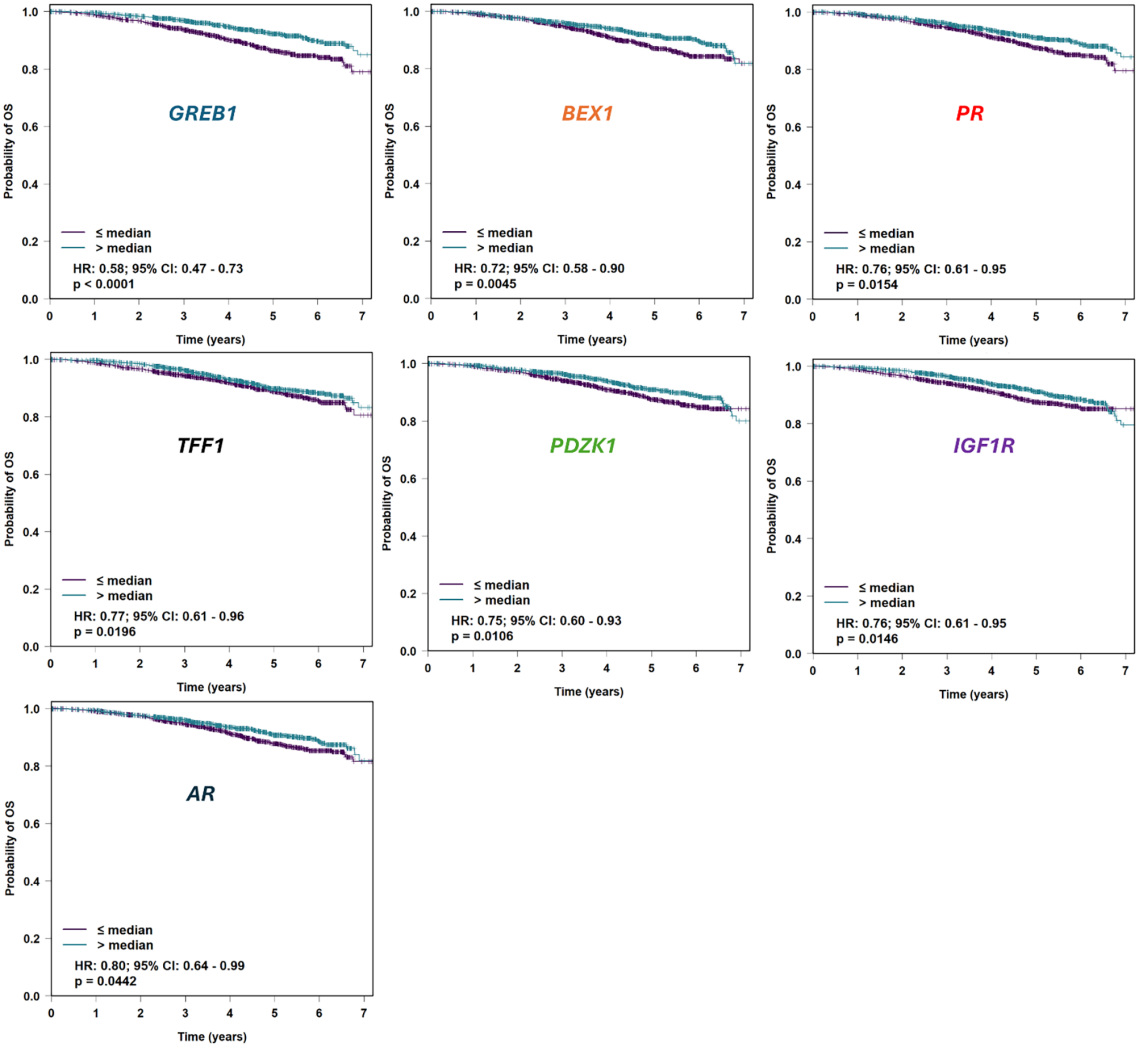
Kaplan Meier survival plots for studied ER‐responsive genes with prognostic significance, extracted from bc‐GenExMiner. OS, overall survival.

Survival analysis of all ER+ RNA‐seq data from bc‐GenExMiner showed longer survival was associated with higher expression of the following seven genes: *GREB1* (*P* < 0.0001), *BEX1* (*P* = 0.004), *PR* (*P* = 0.01), *TFF1* (*P* = 0.01), *PDZK1* (*P* = 0.01), *IGF1R* (*P* = 0.01) and *AR* (*P* = 0.04) (Figure 2). No other genes were found to have prognostic value.

### Proteomic Analysis

#### Correlation to ER

IHC expression data has been correlated with ER expression and the specificity and sensitivity of 21 ER‐regulated biomarkers to ER+ status have been investigated and summarised in Table 2. PR, GREB1 (detailed in Data S1) and GATA3 notably revealed the highest specificity and AUC to ER expression.

**Table 2:** Correlation analysis of oestrogen receptor (ER)‐regulated biomarkers to ER status and ER% in the Nottingham cohort

Biomarker protein	Total n	Biomarker+/ER+	Biomarker+/ER‐	Biomarker−/ER+	Biomarker‐ /ER‐	Specificity to ER+ status	Sensitivity to ER+ status	Area under the curve (AUC)	Correlation to ER *r* (*P*‐value)
PR	3328	2243	24	718	843	97%	76%	0.87	0.5 (<0.001)
GREB1	1491	889	8	451	143	95%	66%	0.80	0.3 (<0.001)
GATA3	1010	435	20	292	263	93%	60%	0.77	0.5 (<0.001)
SSTR2	72	8	2	40	22	92%	17%	0.49	0.2 (<0.001)
IGF1R	1135	117	63	719	236	79%	14%	0.48	0.4 (<0.001)
SLC7A8	1113	189	57	690	177	76%	22%	0.5	0.01 (0.8)
FOXA1	1169	511	79	344	235	75%	60%	0.67	0.3 (<0.001)
AR	1706	956	126	301	323	72%	76%	0.7	0.5 (<0.001)
BCL2	1007	645	77	110	175	69%	85%	0.82	0.5 (<0.001)
SLC39A6	660	215	66	236	143	68%	48%	0.59	0.1 (<0.001)
AGR2	826	564	136	11	115	46%	98%	0.8	0.4 (<0.001)
TFF1	1026	618	171	145	92	35%	81%	0.6	0.2 (<0.001)
BEX1	1124	610	192	221	101	34%	73%	0.53	0.1 (0.04)
PBX1	1189	658	235	204	92	28%	76%	0.56	0.1 (<0.001)
TFF3	1025	97	597	151	180	23%	39%	0.72	0.3 (<0.001)
AGTR1	966	549	199	160	58	23%	77%	0.47	−0.03 (0.3)
ERBB4	1508	856	340	262	50	13%	77%	0.42	−0.1 (<0.001)
ST8SIA6	1197	815	287	72	23	7%	92%	0.43	−0.1 (<0.001)
GLS2	643	421	177	33	12	6%	93%	0.45	−0.07 (0.1)
GFRA1	1144	767	325	35	17	5%	96%	NA^a^	NA^a^
PDZK1	1283	1008	273	2	0	‐	99%	0.46	−0.06 (0.04)

*r* = Pearson's rank correlation coefficient.

^a^ Scores were available in categories (positive/negative); correlation for continuous variables was non‐applicable.

#### Survival analysis

**Figure 3:**
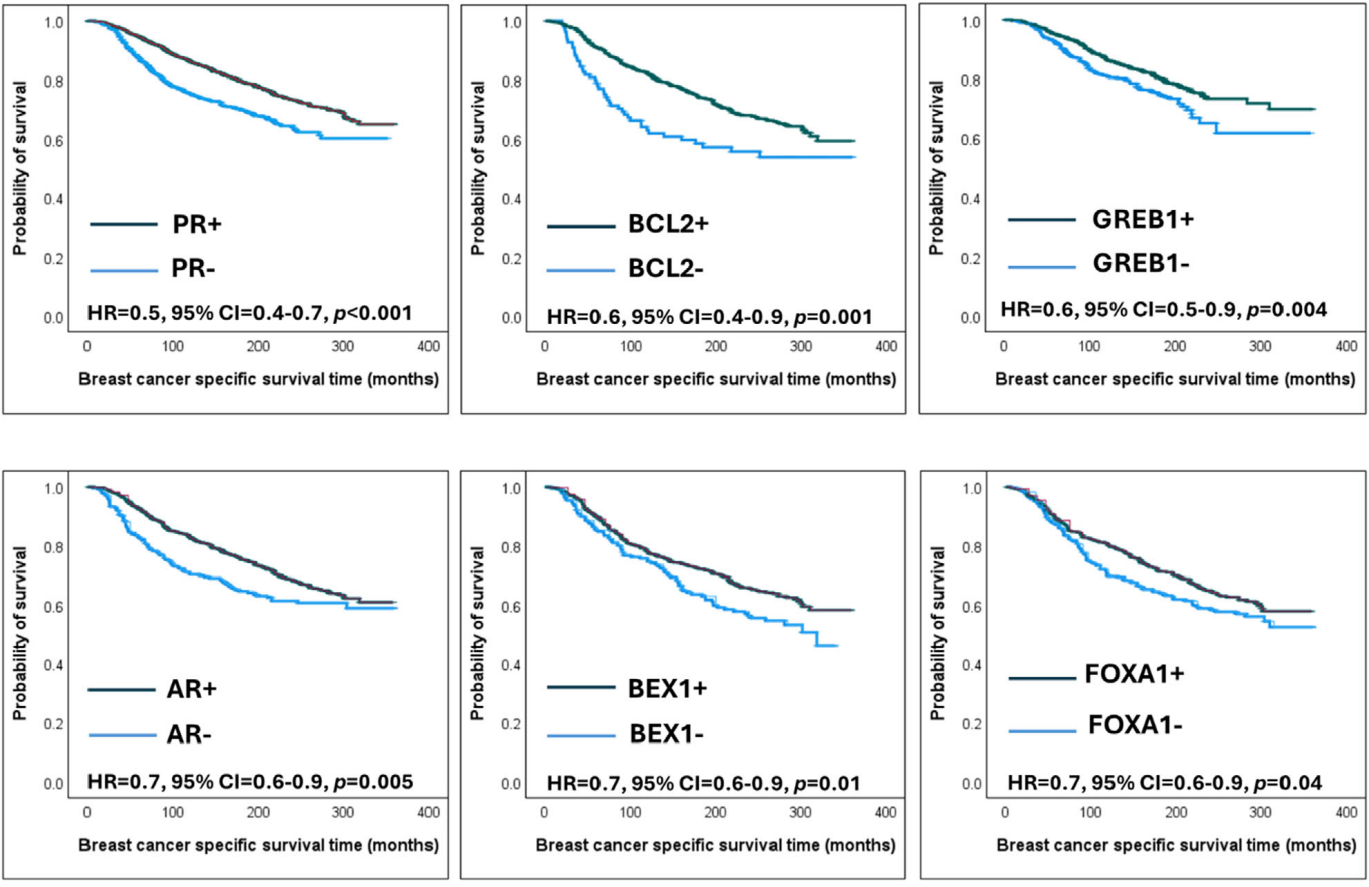
Kaplan Meier survival plots of prognostic oestrogen receptor (ER)‐regulated biomarkers in ER+ Nottingham cohort.

The prognostic significance of ER‐regulated biomarkers was examined using Kaplan–Meier survival analysis in the entire ER+ BC Nottingham cohort. Similar to the transcriptomic results, significant associations with favourable prognosis were observed in ER+ BC expressing PR (*P* < 0.001), GREB1 (*P* = 0.004), AR (*P* = 0.005) and BEX1 (*P* = 0.01). Additionally, longer survival outcomes were associated with higher BCL2 (*P* = 0.001) and FOXA1 (*P* = 0.04) expression (Figure 3).

#### Common genes of transcriptomic and proteomic prognostic significance

To filter the genes of possible clinical significance, we selected the four biomarkers (GREB1, PR, BEX1 and AR) that showed prognostic significance in ER+ BC on both the transcriptomic and proteomic levels. Subsequently, they were further tested for their ability to predict the outcome of ER+ BC patients who were treated with adjuvant ET as the only systemic therapy (patients who received chemotherapy were excluded from the analysis).

#### Endocrine sensitivity biomarkers

Using the Kaplan–Meier Plotter web tool, only *GREB1* was significantly associated with a better response of ER+ BC patients to ET with a prolonged OS (*p* = 0.002). However, biomarkers' protein expression levels in the Nottingham cohort revealed that favourable BCSS outcomes in ET‐treated patients were associated with high PR (*P* < 0.001), high AR (*P* < 0.001) and high GREB1 expression (*P* = 0.004) (Figure 3).

Each significant predictive biomarker was tested in a multivariate Cox Regression model, adjusted for grade, LN metastasis and tumour size. Only GREB1 and PR remained independent predictors of prolonged survival (Table 3A,B). When both GREB1 and PR were added to a multivariate model, both retained statistical significance (Table 3C).

**Table 3:** Four breast cancer‐specific survival multivariate Cox Regression analysis models of endocrine therapy‐treated patients, evaluating the predictive independence of PR (A) and GREB1 (B) biomarkers, individually and in combination (C and D)

	Parameter	Hazard ratio	95% confidence interval	*P*‐value
A	*PR*	0.7	0.6–0.8	<0.001
	Grade	0.5	0.4–0.6	<0.001
	Tumour size	0.6	0.5–0.7	<0.001
	Lymph node metastasis	0.5	0.4–0.6	<0.001
B	*GREB1*	0.7	0.5–0.9	0.009
	Grade	0.3	0.2–0.4	<0.001
	Tumour size	0.6	0.4–0.8	<0.001
	Lymph node metastasis	0.4	0.3–0.5	<0.001
C	*PR*	0.6	0.4–0.8	<0.001
	*GREB1*	0.7	0.5–0.9	0.04
	Grade	0.3	0.2–0.5	<0.001
	Tumour size	0.6	0.4–0.8	0.002
	Lymph node metastasis	0.4	0.3–0.6	<0.001
D	*PR‐GREB1 combined score*	0.4	0.3–0.6	<0.001
	Grade	0.3	0.2–0.5	<0.001
	Tumour size	0.6	0.4–0.8	0.002
	Lymph node metastasis	0.4	0.3–0.6	<0.001

#### Combined PR‐GREB1 expression

Considering the independent prognostic significance of both GREB1 and PR, the ET‐treated BC cohort was stratified into three groups based on the expression of PR and GREB1: both positive, one positive and both negative.

**Figure 4:**
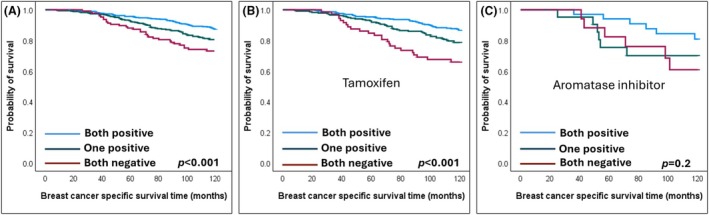
Kaplan Meier survival plots showing favourable breast cancer‐specific survival of endocrine‐treated oestrogen receptor‐positive BC patients associated with protein expression of both PR and GREB1. (**A**) Unstratified endocrine‐treated patients, (**B**) Tamoxifen‐treated and (**C**) Aromatase inhibitor‐treated.

A significant difference was revealed between PR+/GREB1+ tumours and other groups (*P* < 0.001). Tumours with both biomarkers' expression had a more favourable BCSS than BC with only one of them positive (*P* = 0.006) (Figure 4). ET‐treated patients with BC expressing both PR and GREB1 had a 10‐year BC‐specific death risk of 12%, as opposed to 18% for one biomarker positive and 26% when both markers were negative.

**Figure 5:**
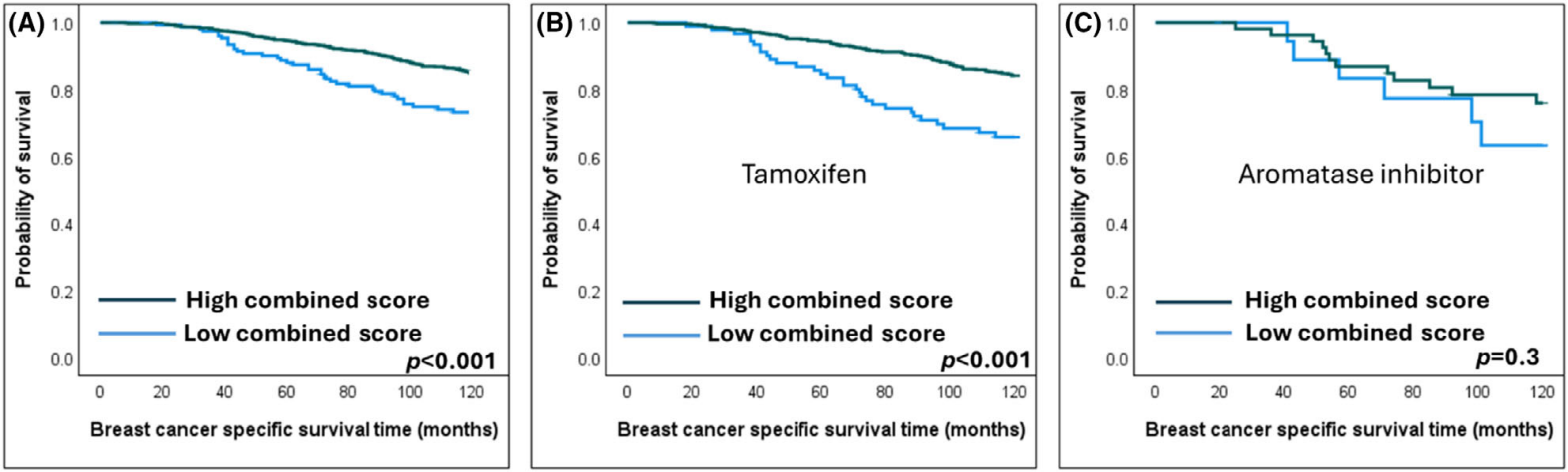
Kaplan Meier survival plots showing favourable breast cancer‐specific survival of endocrine‐treated oestrogen receptor‐positive BC patients associated with high combined score of PR and GREB1. (**A**) Unstratified endocrine‐treated patients, (**B**) Tamoxifen‐treated and (**C**) Aromatase inhibitor‐treated.

Subsequently, a combined score was generated to obtain a signature predictive of better response of ER+ BC patients to ET. Using the regression coefficient (β) of both biomarkers in the multivariate Cox Regression model as follows: [(0.33 × GREB1%) + (0.55 × PR%)], a combined score was created. The resulting score was then stratified into low and high using BCSS in X‐tile software.^56^ A significantly prolonged BCSS was associated with a high PR‐GREB1 score (*P* < 0.001) (Figure 5). The GREB1‐PR score has shown independence as a predictive score in the multivariate survival model with a lower HR than PR or GREB1 alone (HR = 0.4, 95% CI = 0.3–0.6, *p* < 0.001) (Table 3D).

A subgroup analysis was conducted based on the type of ET administered (tamoxifen or aromatase inhibitors). The combined expression of PR and GREB1 significantly predicted favourable patient responses to tamoxifen (*P* < 0.001) but showed no significance in those treated with aromatase inhibitors (Figures 4 and 5).

When PAM50 molecular subtyping was considered, both PR‐GREB1 IHC score (HR = 0.6, 95% CI = 0.4–0.8, *P* = 0.001) and the luminal subtype (vs. non‐luminal types, HR = 0.4, 95% CI = 0.2–0.8, *P* = 0.005) demonstrated comparable significance in predicting response to ET.

#### External validation

This combined score was further validated on the mRNA level in the METABRIC dataset, where the same model was applied to the transcript values to generate a combined *PR‐GREB1* score. The high combined score group was significantly associated with prolonged BCSS compared to the low score group (*P* = 0.005) (Figure S4).

Using KM‐plotter web tool, a combination of the *PR*‐*GREB1* mean expression values was carried out on ER+ ET‐treated patients. The combined expression showed a strong statistical significance (*P* = 0.0003), outperforming that of *GREB1* alone (Figure S5).

## Discussion

Oestrogen regulates BC cell development and progression through activation of ER,^10^, ^57^ which is expressed in approximately two‐thirds of BCs and drives critical biological and therapeutic implications.^58^ Gene expression profiling of BC has demonstrated that ER+ and ER‐ BC are distinct tumours with different pathogenesis, morphology and clinical behaviour.^18^, ^59^ However, the crosstalk between ER and oestrogen in terms of ER signalling and ER‐regulated genes, in addition to associated co‐regulators, is still not fully understood.^36^, ^60^

Although ER expression is the strongest predictor of response to ET, the response of ER+ BC is variable and a proportion of ER+ tumours recur despite ET. Therefore, refinement of the prediction of ER+ to ET is needed.^33^ Several ER‐regulated or related genes have been suggested as potential predictive markers for ET response^33^, ^36^, ^61^; nevertheless, discrepancies across studies and lack of adequate validation on large cohorts and when evaluated in the context of other ER‐regulated genes limited their clinical use. Moreover, a trend toward identifying a set of genes rather than a single gene that could best determine the response to ET was adopted.^12^, ^17^, ^62^, ^63^

In this study, several hundred genes were found to be associated with ER expression at the transcriptomic levels, with some upregulated and some downregulated in ER+ BC. We focused on the upregulated DEGs related to ER‐positivity that can serve as markers for ET response or targets for therapy. To narrow down the number of genes to be assessed, the top DEGs were evaluated for their biological and clinical significance from previous studies and 21 genes were selected.^17^, ^18^, ^32^, ^33^, ^34^, ^35^, ^36^, ^37^, ^38^, ^39^, ^40^, ^41^, ^42^, ^43^, ^44^, ^45^, ^46^, ^47^, ^64^ Of the 21 selected genes, 20 had protein expression data in the Nottingham cohort, while one (GREB1) that did not have IHC data was stained in this study. Further analyses of the prognostic values of these genes in ER+ BC, at both transcriptomic and protein levels, showed that four biomarkers (PR, GREB1, AR and BEX1) maintained their prognostic significance, with only two genes (PR and GREB1) maintaining their independent prognostic significance in ER+ BC patients who received ET.

The regulation of ER‐dependent genes, such as PR, has been extensively studied.^18^, ^32^, ^33^, ^34^, ^35^, ^36^, ^65^ While ER is a well‐established and reliable predictor of response to ET, the role of PR remains a topic of debate.^48^ Although the ASCO/CAP recommends routine evaluation of PR in BC,^66^ the UK Royal College of Pathologists (RCPath) considers PR testing in BC to be optional.^67^ The findings of the present study further highlight the predictive value of PR in ET response and support its inclusion in routine IHC testing for BC, particularly in laboratories that currently assess ER alone.

There is a reciprocal regulatory interaction between PR and ER expression, with ER regulating PR expression and PR, in turn, influencing ER expression.^48^ The presence of PR indicates that the ERα pathway is active.^68^ Additionally, PR not only serves as an ERα‐induced gene target but also as an ERα‐associated protein that influences its behaviour. PR cooperates with ERα to guide ERα chromatin binding events within BC cells, resulting in a distinct gene expression associated with favourable clinical outcomes.^69^, ^70^ Research has demonstrated that patients with tumours that are ER+/PR+ benefit more from adjuvant tamoxifen therapy compared to those with ER+/PR‐ tumours.^71^ Although only ER is currently utilised as a widely accepted predictive marker for hormonal therapy, recent studies have suggested that PR levels are directly and positively linked to treatment response. Elevated PR levels have been shown to significantly and independently correlate with an increased likelihood of response to tamoxifen.^48^, ^72^, ^73^

Growth Regulating Oestrogen Receptor Binding 1 (GREB1) was first described by Ghosh *et al.* ^34^ as an oestrogen‐related gene. It has been found that *GREB1* is involved in the regulation of oestrogen‐induced stimulation of cell proliferation and is activated upon stimulation by oestrogen.^34^, ^74^ *GREB1*, described as an early ER‐regulated gene, was found to be correlated to hormone expression in reproductive tissues.^75^, ^76^, ^77^, ^78^ It has been shown to be highly expressed in ER+ (MCF‐7, T‐47D and BT‐474) in contrast to ER‐ cell lines (MDA‐MB 231 and SUM 225).^74^, ^79^ *GREB1* has a role in regulating the transcription of ER‐target genes, as exposed upon *GREB1* knockdown in MCF‐7 cells where a significant down expression was noticed in ER‐regulated genes.^80^ Additionally, methylation‐mediated *GREB1* downregulation was identified in tamoxifen‐resistant BC, suggesting the possibility of targeting GREB1 for better understanding of BC resistance and providing more effective treatment options.^81^, ^82^

However, *GREB1* as an ER‐target gene lacked validation on a large, well‐characterised cohort and the correlation with patient outcome and response to therapy was insufficient, which limits its clinical application. In this study, IHC analysis of GREB1 expression in a large BC cohort revealed very high specificity of this biomarker to ER positivity. Similar results were reported by Mohammed and colleagues.^80^ A study involving 338 ER+ BC cases demonstrated a high specificity (91%) of GREB1 to ER status, which also proved to be an independent prognostic biomarker. Furthermore, ER‐ cell lines lacked GREB1 expression, while it was detected in ER+ cell lines and IHC validation was carried out on 105 ER+ and 87 ER‐ cases in a previous report.^79^ Patients with high ER‐GREB1 co‐expressing tumours had favourable survival outcomes and response to ET when compared to ER+ BC lacking GREB1 expression, highlighting the role of GREB1 for a functional ER pathway.^80^

AR has been reported to be highly expressed in ER+ BC and associated with favourable prognosis on both the protein and transcriptomic levels,^49^ in line with our study. However, AR is widely expressed in BC molecular subtypes and known to be expressed in ER‐ special histological subtypes.^83^, ^84^ Moreover, the role of AR in ER+ BC remains controversial.^85^ AR was reported to have an antiproliferative effect, while it was also suggested to be involved in resistance to ET.^86^, ^87^ Despite the low specificity of BEX1 to ER+ status, we noticed a consistently favourable prognostic significance with its association with ER+ BC on the mRNA and protein levels. BEX1 expression was evaluated in BC molecular subtypes, where all samples with BEX1 overexpression were ER+ in a previous report.^88^ It has been suggested as a pro‐apoptotic gene^89^; however, the role of *BEX1* in BC is not yet understood.^90^

The two independent predictive biomarkers of all investigated in the current study were PR and GREB1. This was reflected in the 10‐year BC‐specific death risk of 12%, compared to 26% in BC with positive versus negative PR and GREB1 expression. Their combined expression carried a favourable ET response over a single biomarker expression. Furthermore, their combined score provided a robust independent predictive score in ET‐treated patients.

We propose that the combination of PR and GREB1 may serve as a potential surrogate marker for predicting ET responsiveness in BC. Based on our findings, additional testing for PR and GREB1 in ER+ BC could help identify patients who are likely to respond to ET, potentially sparing them from more aggressive treatment strategies. Once the necessary qualification, verification and validation criteria for clinical translation are met, these biomarkers could be integrated into standard clinical workflows.

Although this study involved large cohorts of BC patients, it has certain limitations. Its retrospective design necessitates confirmation of the findings through prospective randomised clinical studies. Such validation is essential before clinical implementation. While functional validation was beyond the scope of this study, we recommend future *in vitro* studies to elucidate the mechanistic and biological pathways associated with combined PR and GREB1 expression. Comprehensive molecular investigations would further strengthen the biological rationale for the role of PR and GREB1 in ET resistance. Moreover, this study focused exclusively on genes upregulated by ER. However, ER also exerts repressive effects on gene transcription, a function that is less well characterised. The role of downregulated genes should therefore be explored further, as they may also play a significant role in therapy response. In conclusion, targeting ER downstream signalling would provide a better understanding of ER+ BC, offering innovative treatment options for patients resistant to ET. Compared to other known ER‐regulated genes, PR and GREB1 are potential therapeutic targets for ER+ BC that proved to be ER‐specific and robust prognostic and predictive biomarkers, alone and in combination.

## Author contributions

SM took the lead in writing the manuscript, biomarker scoring, data analysis and interpretation. NA and AS contributed to biomarker scoring, data analysis and article review. NA, AI, MT and NPM helped with data interpretation and article review. ER conceived and planned the presented idea, data interpretation and article review.

## Conflicts of interest

The authors have declared no conflicts of interest.

## Supporting information


**Table S1.** Clinicopathological characteristics of the Nottingham cohort.
**Table S2.** Details of antibodies used in the study.
**Figure S1.** Shows examples of immunohistochemical expression of key biomarkers studied. (***A***). *ER (x10)*, (***B***) *PR (x10)*, (***C***) *GREB1 (x20)*, (***D***) *GATA3 (x10)*, (***E***) *AR*, (***F***). *BCL2 (x20)*, (***G***) *GFRA1 (x20)*, (***H***) *AGR2 (x20)*, (***I***) *IGF1R (x20)*, (***J***) *GLS2 (x20)*, (***K***) *PBX1(x20)*, (***L***) *SSTR2 (x20)*, (***M***) *PDZK1 (x20), and* (***N***) *SLC7A8 (x20)*.
**Figure S2.** Heatmap showing differential expression of ER‐regulated genes across breast cancer cases stratified by ER status.
**Table S3.** Shows the Log2 Fold Change and adjusted p‐value (FDR) of differential gene expression analysis between oestrogen receptor‐positive and oestrogen receptor‐negative breast cancers.
**Figure S3.** Kaplan Meier survival plots were carried out on endocrine‐treated oestrogen receptor‐positive breast cancer patients, showing favourable breast cancer‐specific and distant metastasis‐free survival outcomes with positive PR (**A** and **B**), AR (**C** and **D**), and GREB1 (**E** and **F**).
**Figure S4.** Kaplan Meier survival plot of endocrine‐treated oestrogen receptor‐positive breast cancer patients using the external validation cohort (METABRIC) show favourable breast cancer specific survival associated with the high combined expression of *PR*‐*GREB1*, compared to low combined expression.
**Figure S5.** Kaplan Meier survival plots of endocrine‐treated oestrogen receptor‐positive breast cancer patients using Kaplan Meier Plotter show favourable overall survival associated with the combined mean expression of *PGR*‐*GREB1*, compared to GREB1 alone.
**Figure S6.** Immunohistochemical expression of GREB1 showing diffuse cytoplasmic positivity in oestrogen receptor (ER)‐positive breast cancer (BC) (**A**) and absence of expression in ER‐negative BC (**B**).
**Figure S7.** ROC curve showing the sensitivity and specificity of GREB1 to ER status.

## Data Availability

The data presented in the current study are available upon reasonable request.
