# Supplementary material for: Outcome prediction of oestrogen receptor‐positive breast cancer based on a panel of oestrogen receptor‐regulated genes
Source: Histopathology. 2025 Sep 12;87(6):842–55. doi: 10.1111/his.15557 (PMC12605772; doi:10.1111/his.15557)
Supplement: Supplementary file 1 — Table S1. Clinicopathological characteristics of the Nottingham cohort. Table S2. Details of antibodies used in the study. Figure S1. Shows examples of immunohistochemical expression of key biomarkers studied. ( A ). ER (x10), ( B ) PR (x10), ( C ) GREB1 (x20), ( D ) GATA3 (x10), ( E ) AR, ( F ). BCL2 (x20), ( G ) GFRA1 (x20), ( H ) AGR2 (x20), ( I ) IGF1R (x20), ( J ) GLS2 (x20), ( K ) PBX1(x20), ( L ) SSTR2 (x20), ( M ) PDZK1 (x20), and ( N ) SLC7A8 (x20). Figure S2. Heatmap showing differential expression of ER‐regulated genes across breast cancer cases stratified by ER status. Table S3. Shows the Log2 Fold Change and adjusted p‐value (FDR) of differential gene expression analysis between oestrogen receptor‐positive and oestrogen receptor‐negative breast cancers. Figure S3. Kaplan Meier survival plots were carried out on endocrine‐treated oestrogen receptor‐positive breast cancer patients, showing favourable breast cancer‐specific and distant metastasis‐free survival outcomes with positive PR (A and B), AR (C and D), and GREB1 (E and F). Figure S4. Kaplan Meier survival plot of endocrine‐treated oestrogen receptor‐positive breast cancer patients using the external validation cohort (METABRIC) show favourable breast cancer specific survival associated with the high combined expression of PR‐GREB1, compared to low combined expression. Figure S5. Kaplan Meier survival plots of endocrine‐treated oestrogen receptor‐positive breast cancer patients using Kaplan Meier Plotter show favourable overall survival associated with the combined mean expression of PGR‐GREB1, compared to GREB1 alone. Figure S6. Immunohistochemical expression of GREB1 showing diffuse cytoplasmic positivity in oestrogen receptor (ER)‐positive breast cancer (BC) (A) and absence of expression in ER‐negative BC (B). Figure S7. ROC curve showing the sensitivity and specificity of GREB1 to ER status. [file HIS-87-842-s001.docx]

**Supplementary Table 1.** Clinicopathological characteristics of the Nottingham cohort.

| **Characteristics** | **N** | **%** |
| --- | --- | --- |
| **Age at diagnosis (Years)**  < 50  ≥ 50 | 1353  2869 | 32  68 |
| **Tumour size (cm)**  < 2  ≥ 2 | 2475  1741 | 59  41 |
| **Tumour Grade**  1  2  3 | 757  1584  1881 | 18  37  45 |
| **Mitotic count**  1  2  3 | 1864  817  1541 | 44  19  37 |
| **Nuclear pleomorphism**  1  2  3 | 148  1516  2558 | 3  36  61 |
| **Tubule formation**  1  2  3 | 325  1329  2568 | 8  31  61 |
| **Nottingham Prognostic Index**  Good Prognostic Group  Moderate Prognostic Group  Poor Prognostic Group | 1522  2080  613 | 36  49  15 |
| **Axillary nodal status**  Negative  Positive | 2698  1523 | 64  36 |
| **Lymph node stage**  1 (Negative)  2 (1-3 positive)  3 (>3 positive) | 2698  1157  366 | 64  27  9 |
| **Lymphovascular Invasion**  Negative  Positive | 3001  1221 | 71  29 |
| **Oestrogen receptor**  Negative  Positive | 961  3145 | 23  77 |
| **Progesterone receptor**  Negative  Positive | 1565  2268 | 41  59 |
| **Human epidermal growth factor receptor 2**  Negative  Positive | 3376  476 | 88  12 |
| **Endocrine therapy**  No  Yes | 2108  2066 | 50  50 |
| **Chemotherapy**  No  Yes | 3168  1051 | 75  25 |

**Supplementary Table 2.** Details of antibodies used in the study.

| **Antibody** | **Subcellular localisation** | **Source** | **Clone/Product code** | **Dilution** |
| --- | --- | --- | --- | --- |
| **GREB1** | Cytoplasmic | Abcam | ab72999 | 1/100 |
| **PR** | Nuclear | Dako | clone 636 | 1/100 |
| **GATA3** | Nuclear | Abcam | EPR16651 | 1/250 |
| **FOXA1** | Nuclear | Abcam | ab40868 | 1/2000 |
| **BCL2** | Cytoplasmic | Dako | Clone 124 | 1/400 |
| **TFF1** | Cytoplasmic | Thermofischer | PA1-28875 | 1/50 |
| **ERBB4** | Cytoplasmic | Thermofischer | RB-905 | 1/50 |
| **TFF3** | Cytoplasmic | Abcam | EPR3974 | 1/100 |
| **AGTR1** | Cytoplasmic | Thermofischer | PA5-20812 | 1/25 |
| **GFRA1** | Cytoplasmic | Abcam | ab8026 | 1/50 |
| **BEX1** | Cytoplasmic | Invitrogen | PA5-24192 | 1/50 |
| **SLC39A6** | Cytoplasmic | LSBio | AA170320 | 1/200 |
| **AGR2** | Cytoplasmic | Novus Biologicals | NBP1-40630 | 1/5000 |
| **IGF1R** | Cytoplasmic | Cell Signalling Technology | 3027 | 1/1000 |
| **GLS2** | Cytoplasmic | Abcam | EP7212 | 1/50 |
| **PBX1** | Nuclear | Abnova | clone 4A2 | 1/50 |
| **AR** | Nuclear | Dako | M3562 | 1/50 |
| **ST8SIA6** | Cytoplasmic | Sigma Aldrich | HPA011635 | 1/100 |
| **SSTR2** | Cytoplasmic | Invitrogen | PA3-109 | 1/2500 |
| **PDZK1** | Cytoplasmic | Abcam | ab121248 | 1/100 |
| **SLC7A8** | Cytoplasmic | Abcam | EPR17573 | 1/50 |


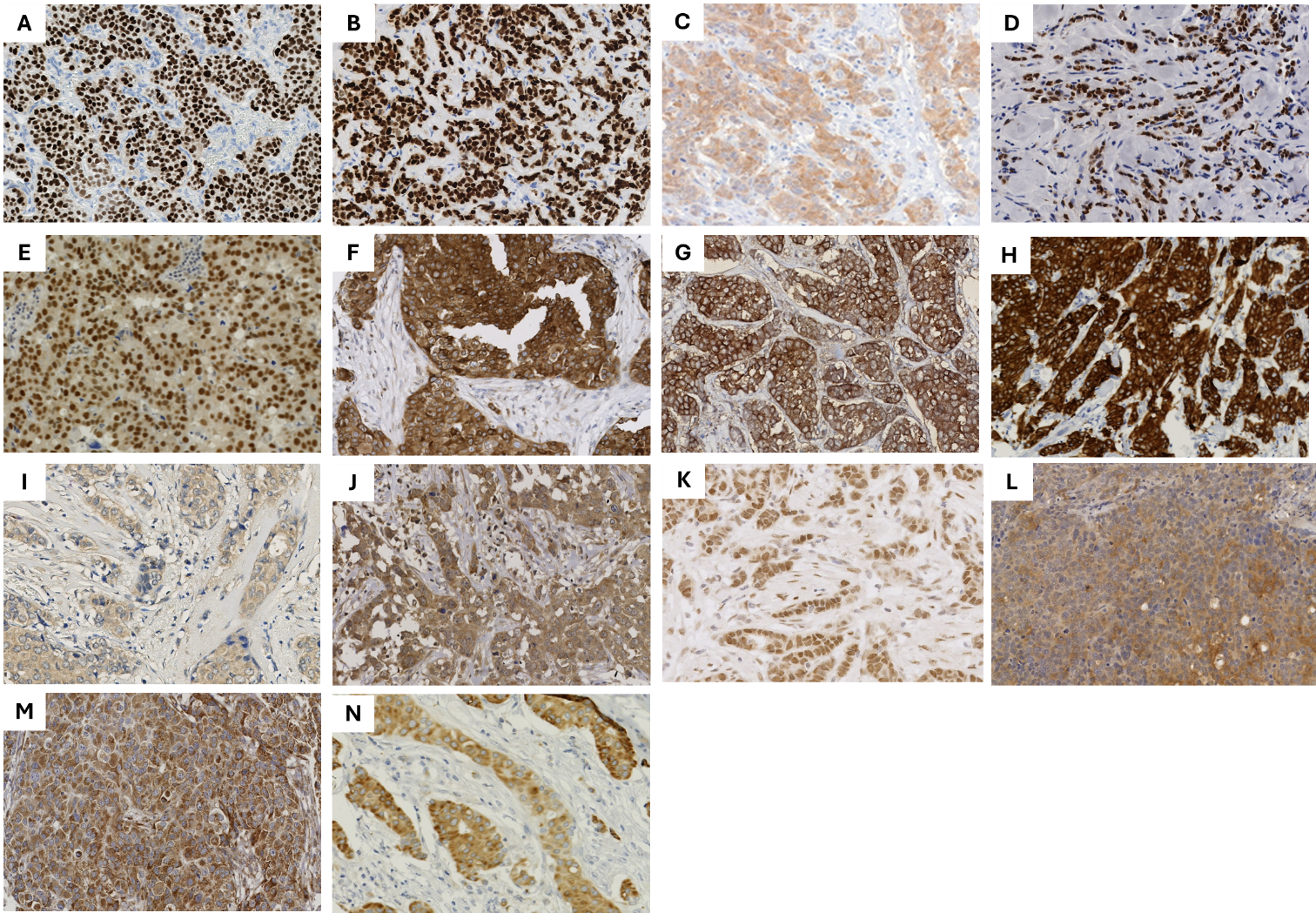


**Supplementary Figure 1**. Shows examples of immunohistochemical expression of key biomarkers studied. **A**. ER (x10), **B**. PR (x10), **C**. GREB1 (x20), **D**. GATA3 (x10), **E**. AR, **F**. BCL2 (x20), **G**. GFRA1 (x20), **H**. AGR2 (x20), **I.** IGF1R (x20), **J**. GLS2 (x20), **K**. PBX1(x20), **L**. SSTR2 (x20), **M**. PDZK1 (x20), and **N**. SLC7A8 (x20).


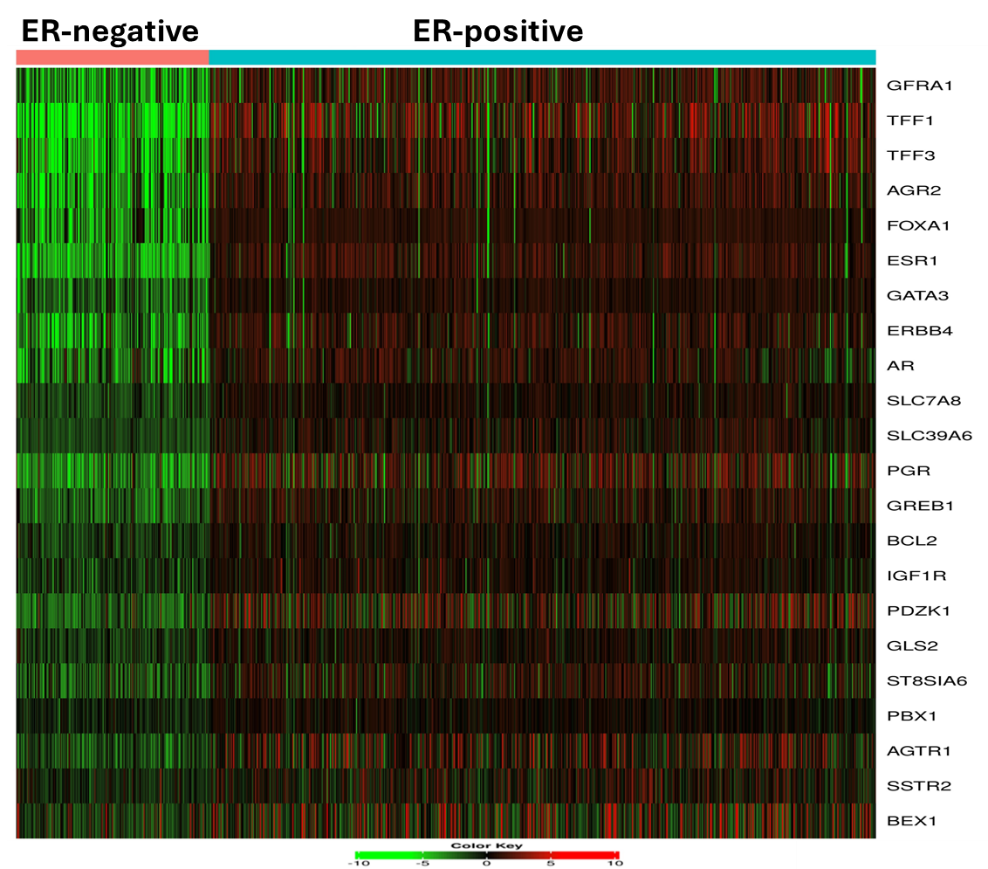


**Supplementary Figure 2**. Heatmap showing differential expression of ER-regulated genes across breast cancer cases stratified by ER status.

**Supplementary Table 3.** Shows the Log2 Fold Change and adjusted p-value (FDR) of differential gene expression analysis between oestrogen receptor-positive and oestrogen receptor-negative breast cancers.

| **Gene symbol** | **Gene name** | **Log2 Fold Change** | **FDR** |
| --- | --- | --- | --- |
| ***TFF1*** | Trefoil Factor 1 | 7.71979 | 8.13E-47 |
| ***TFF3*** | Trefoil Factor 3 | 5.854794 | 9.89E-48 |
| ***AGR2*** | Anterior gradient protein 2 | 5.803343 | 1.41E-70 |
| ***GFRA1*** | GDNF family receptor alpha 1 | 5.182119 | 1.25E-51 |
| ***PR*** | Progesterone receptor | 4.823276 | 5.59E-35 |
| ***ERBB4*** | Erb-B2 Receptor Tyrosine Kinase 4 | 4.811146 | 7.91E-53 |
| ***FOXA1*** | Forkhead box protein A1 | 4.498973 | 4.40E-74 |
| ***PDZK1*** | PDZ Domain Containing 1 | 3.642174 | 8.76E-28 |
| ***GATA3*** | GATA Binding Protein 3 | 3.617467 | 3.88E-96 |
| ***GREB1*** | Growth Regulating Oestrogen Receptor Binding 1 | 3.57337 | 1.27E-62 |
| ***AR*** | Androgen receptor | 3.514199 | 5.00E-37 |
| ***AGTR1*** | Angiotensin II receptor type 1 | 2.856085 | 5.10E-25 |
| ***ST8SIA6*** | ST8 alpha-N-acetyl-neuraminide alpha-2,8-sialyltransferase 6 | 2.74455 | 3.74E-34 |
| ***SLC39A6*** | Solute Carrier Family 39 Member 6 | 2.613229 | 4.40E-74 |
| ***SLC7A8*** | Solute Carrier Family 7 Member 8 | 2.394073 | 2.39E-68 |
| ***BCL2*** | B-Cell Lymphoma 2 | 2.054509 | 4.62E-52 |
| ***GLS2*** | Glutaminase 2 | 1.928512 | 2.08E-43 |
| ***IGF1R*** | Insulin-like growth factor 1 receptor | 1.868924 | 2.86E-40 |
| ***BEX1*** | Brain expressed X-linked 1 | 1.524516 | 4.56E-07 |
| ***SSTR2*** | Somatostatin receptor 2 | 1.127273 | 1.04E-11 |
| ***PBX1*** | PBX homeobox 1 | 1.095324 | 7.07E-32 |


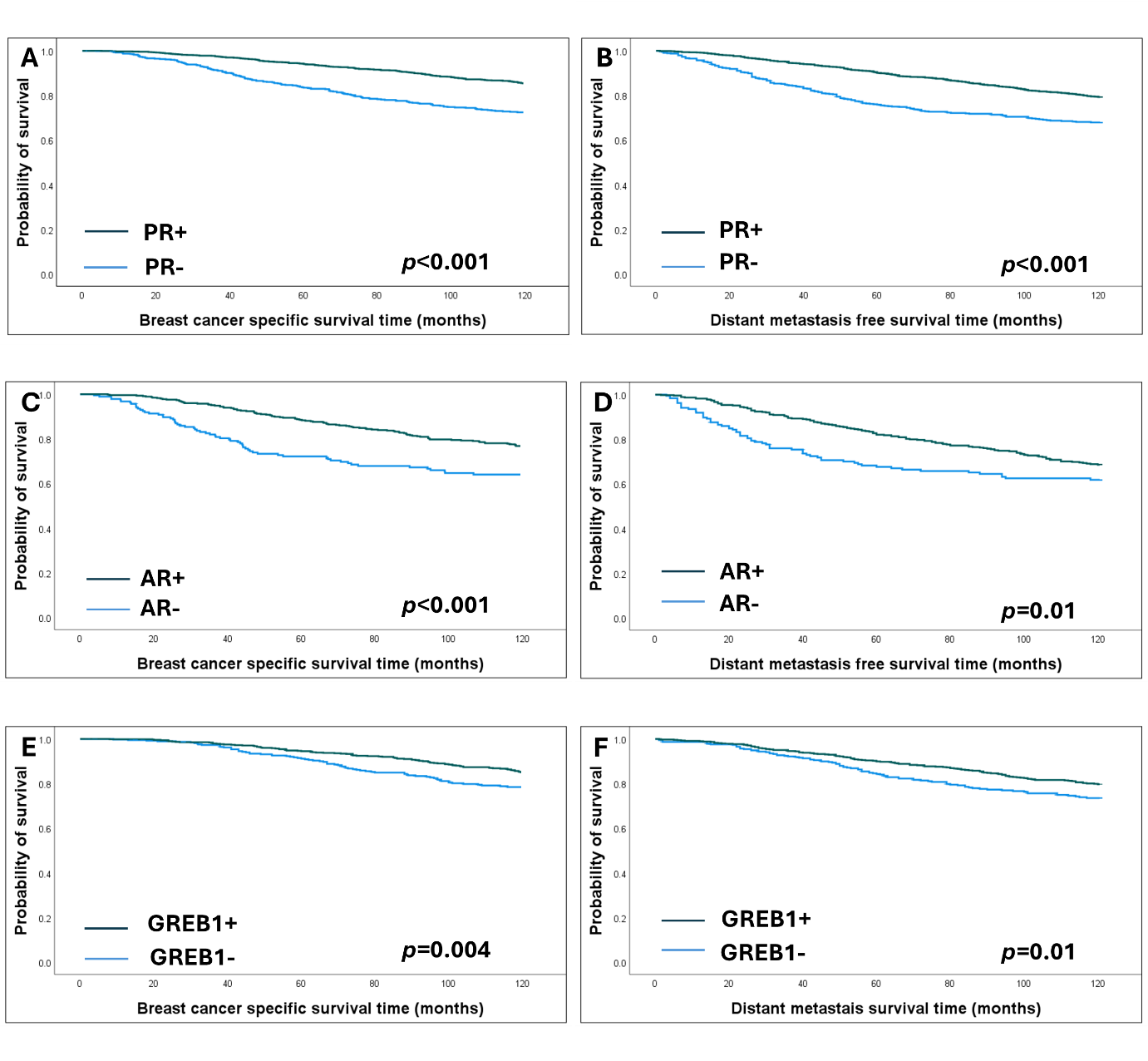


**Supplementary Figure 3.** Kaplan Meier survival plots were carried out on endocrine-treated oestrogen receptor-positive breast cancer patients, showing favourable breast cancer-specific and distant metastasis-free survival outcomes with positive PR (A and B), AR (C and D), and GREB1 (E and F).


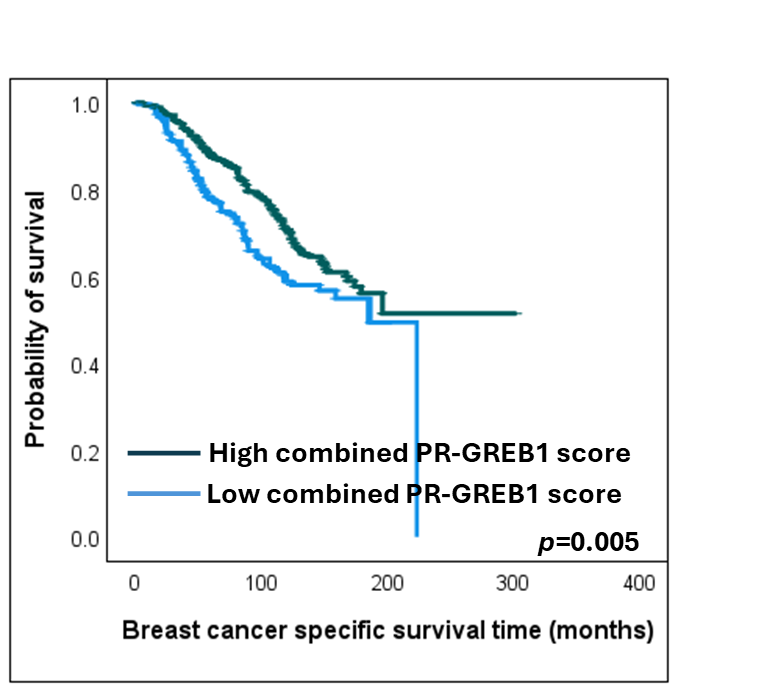


**Supplementary Figure 4.** Kaplan Meier survival plot of endocrine-treated oestrogen receptor-positive breast cancer patients using the external validation cohort (METABRIC) show favourable breast cancer specific survival associated with the high combined expression of PR-GREB1, compared to low combined expression.


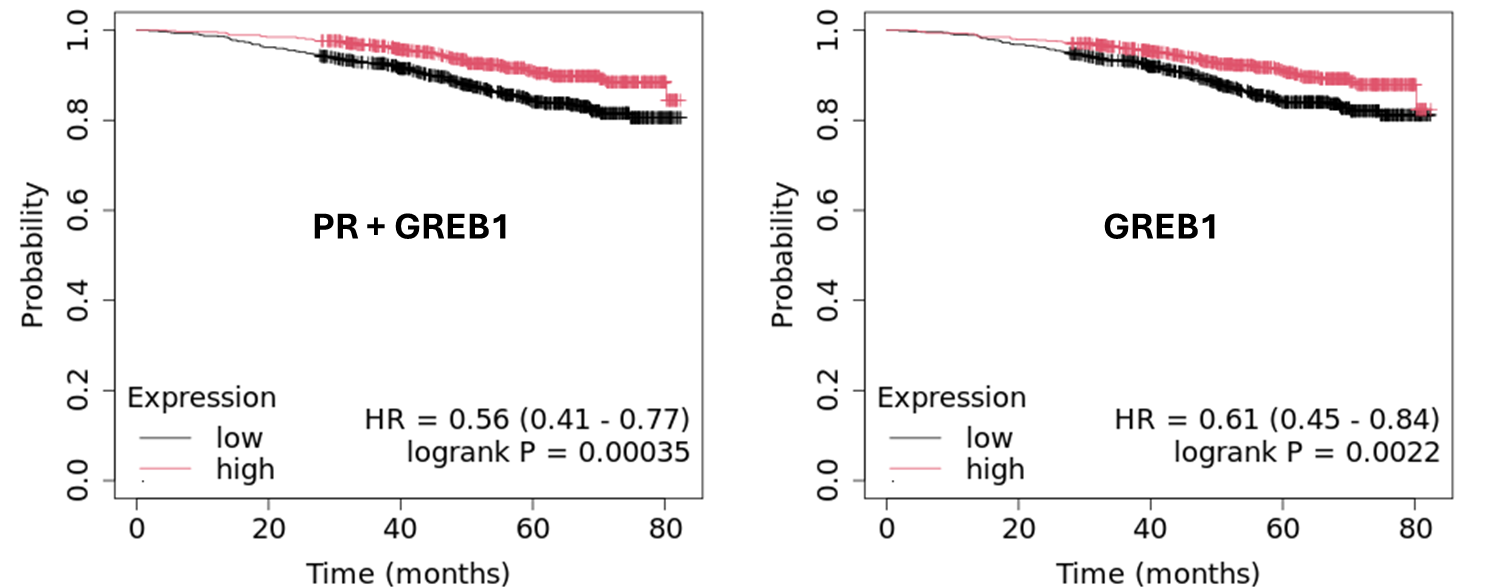


**Supplementary Figure 5.** Kaplan Meier survival plots of endocrine-treated oestrogen receptor-positive breast cancer patients using Kaplan Meier Plotter show favourable overall survival associated with the combined mean expression of PGR-GREB1, compared to GREB1 alone.

**Supplementary data**

GREB1 expression was noted in 60% of total IHC-stained cases in the Nottingham cohort and 66% of the total ER+ BC cases (**Supplementary Figure 6**). GREB1 yielded a 95% specificity to ER+ BC, with only 8 cases being GREB1 positive in the absence of ER expression (AUC=0.8) (**Supplementary Figure 7**), which was the second highest specificity (after PR) compared to other ER-regulated biomarkers. A significant positive linear correlation between ER and GREB1 percentage of IHC expression (r=0.3, *p*<0.001) as well as between PR and GREB1 (r=0.4, *p*<0.001) was observed. Within ER+/PR+ tumours, positive GREB1 expression was noted in 72% of them.


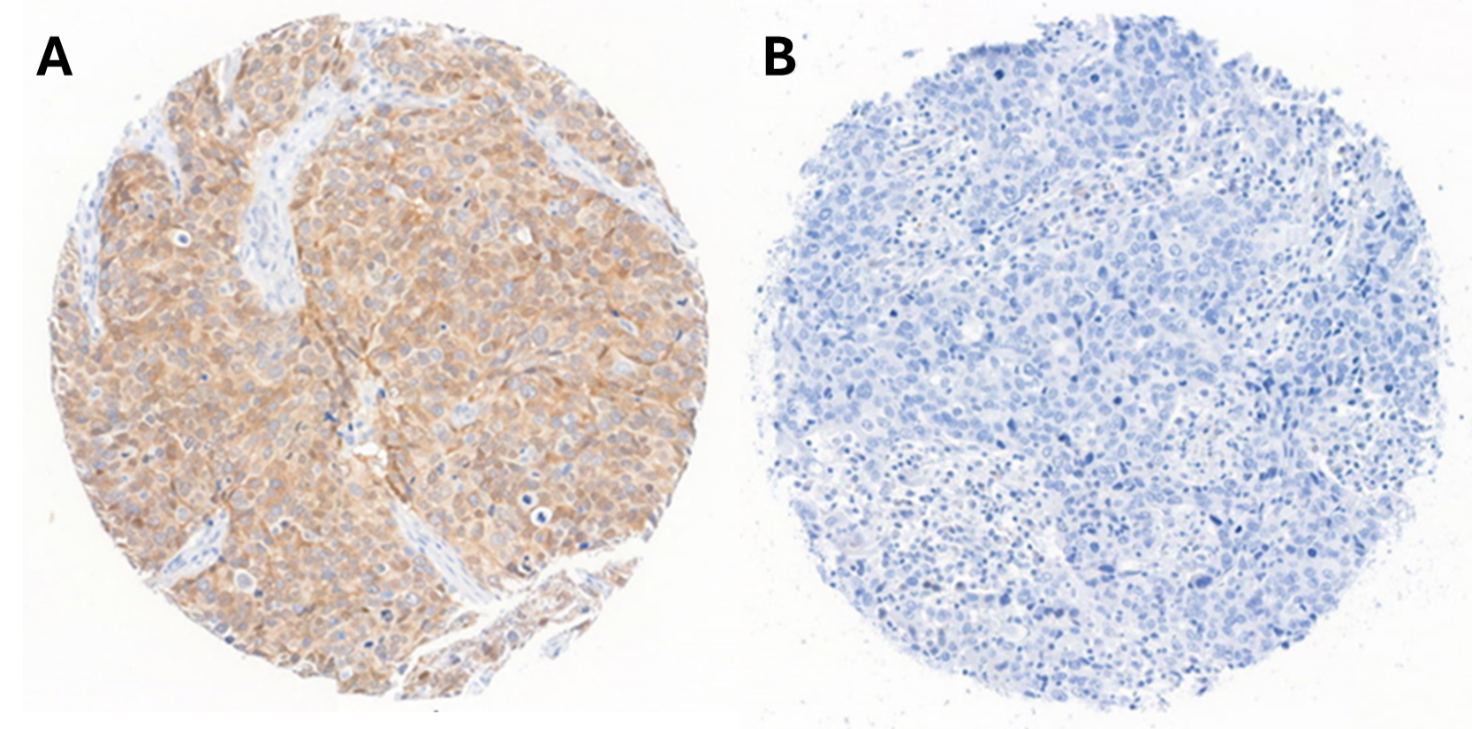


**Supplementary Figure 6.** Immunohistochemical expression of GREB1 showing diffuse cytoplasmic positivity in oestrogen receptor (ER)-positive breast cancer (BC) (A) and absence of expression in ER-negative BC (B).


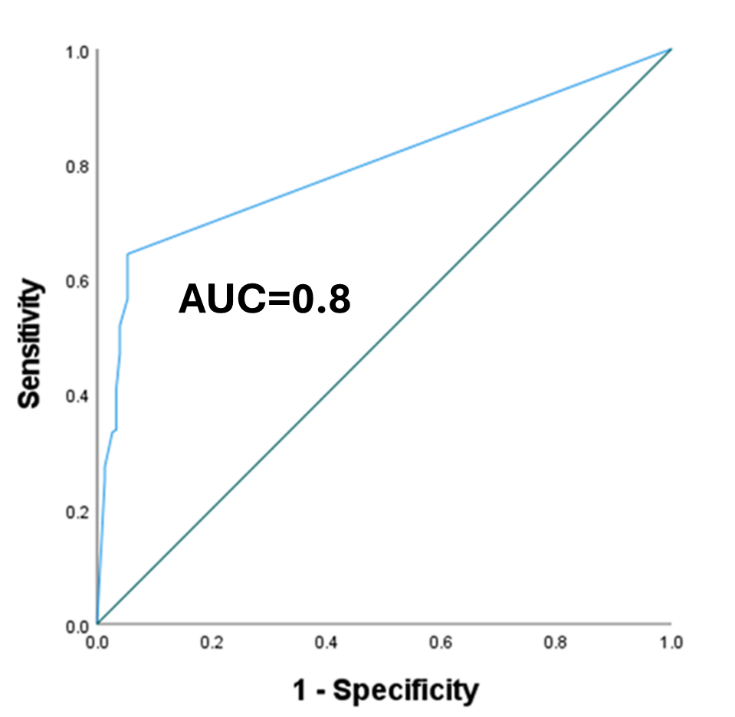


**Supplementary Figure 7.** ROC curve showing the sensitivity and specificity of GREB1 to ER status.
